# A Case of Bilateral Galeazzi Fracture-Dislocations

**DOI:** 10.7759/cureus.17491

**Published:** 2021-08-27

**Authors:** Andreas Panagopoulos, Konstantina Solou, Irini Tatani, Antonis Kouzelis, Zinon Kokkalis

**Affiliations:** 1 Orthopaedic Department, University of Patras, Patras, GRC

**Keywords:** bilateral, galeazzi, fracture-dislocation, internal fixation, druj stability

## Abstract

Bilateral Galeazzi fracture-dislocations are extremely rare injuries and only a few case reports have been described so far in the literature. Herein, we report the case of a 31-year-old bicyclist who* *sustained bilateral Galeazzi fracture-dislocations after a collision with a car. Both radial shaft fractures were simple (AO type 22-A2.3), at the same level (Type I: <7.5 cm from the joint line), and without severe comminution having their apex located dorsally. Internal fixation was accomplished in both fractures with an 8-hole, 3.5-mm locking plate; the stability of the distal radioulnar joint (DRUJ) was assessed with several intraoperative tests and found to be stable so that no additional stabilization was necessary. Postoperatively, the forearms were immobilized in a long forearm cast for four weeks. At the last follow-up evaluation, six years postoperatively, the patient was pain-free, had a full range of motion with a total Mayo wrist score of 95 in both wrists. Bilateral Galeazzi fracture-dislocations are rare injuries requiring proper radial fracture management and thorough assessment of DRUJ stability.

## Introduction

Galeazzi fracture-dislocation is a fracture of the distal third of the radius shaft, associated with distal radioulnar joint (DRUJ) dislocation [1]. This fracture pattern was first reported by Cooper in 1822, but it was Riccardo Galeazzi, who in 1934 presented a series of 18 patients and described the incidence, pathophysiology, and treatment of those injuries [2]. Galeazzi fractures account for 7% of adult forearm fractures (3% in children) and usually occur by axial loading of the outstretched arm with pronation or supination of the wrist, which determines the subsequent angulation of the fracture (i.e., apex volar in supination) [2,3]. Type I fractures, located at the distal third of the radius shaft [4] or more specifically within 7.5 cm from its articular surface [5], demonstrate a higher incidence of DRUJ instability, requiring DRUJ stabilization in 53% [4] to 55% [5] of the patients. This fracture has been well known for its consequences to DRUJ stability and has been named “fracture of necessity” to clarify with early operative treatment, in terms of radial shaft fracture fixation that can reduce the dislocated ulna head without any other intervention; in cases of an unstable DRUJ, as judged intraoperatively, transfixation pinning, fixation of the ulna styloid fracture or rarely, open reduction of the joint are required [6]. Bilateral Galeazzi fracture-dislocations are extremely rare and only a few case reports have been described in the literature [7-10].

The aim of this article is to present a case of bilateral Galeazzi fracture-dislocations in a 31-year-old male, managed successfully with bilateral anatomical plate fixation of the radial shaft fracture without DRUJ stabilization. 

The patient was informed that data concerning the case would be submitted for publication, and he provided written consent.

## Case presentation

**Figure 1:**
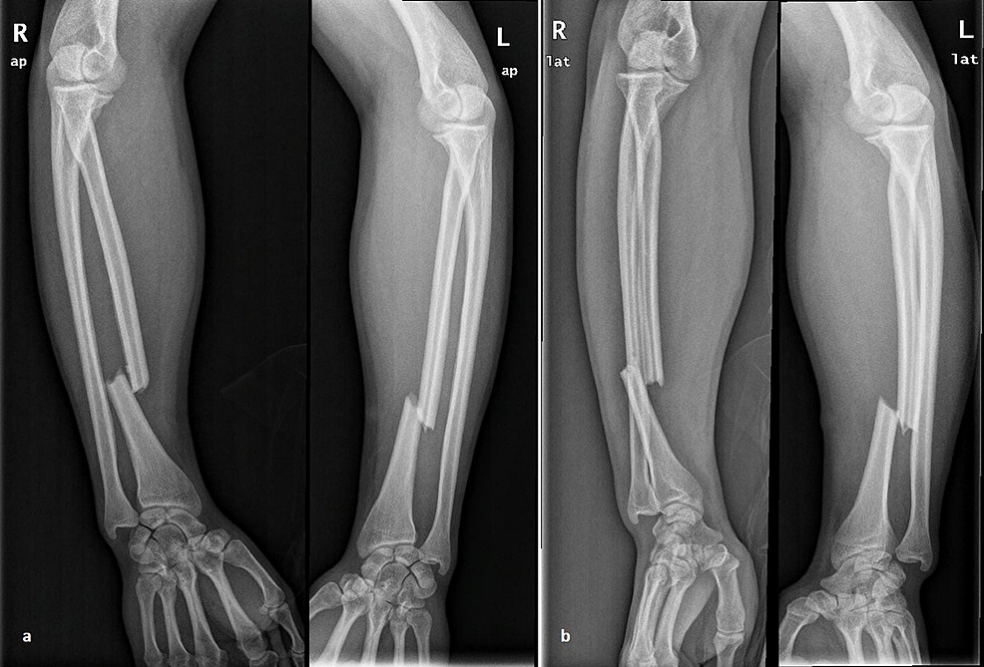
Preoperative radiographs. Preoperative anteroposterior (a) and lateral (b) radiographs of both forearms demonstrating bilateral, almost identical, Galeazzi fracture-dislocations. Both radial fractures were simple, located <7.5 cm from the joint.

**Figure 2:**
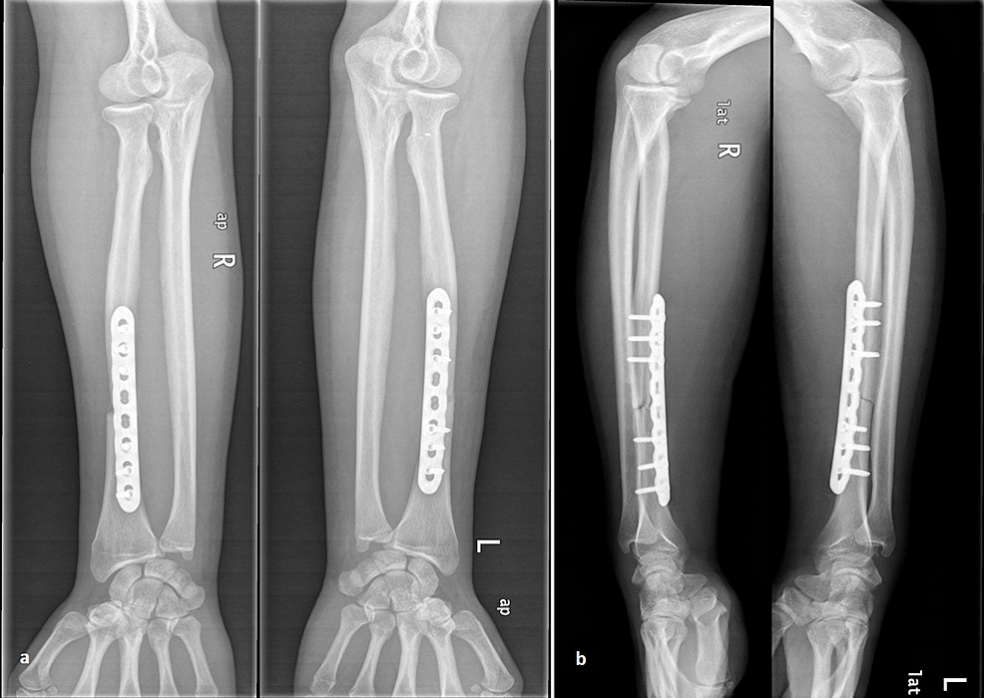
Postoperative radiographs. Postoperative anteroposterior (a) and lateral (b) radiographs of both forearms after removal of the splints (at one month). There is a normal union process of the radial shaft fractures and no evidence of DRUJs’ instability. DRUJ: Distal radioulnar joint.

**Figure 3:**
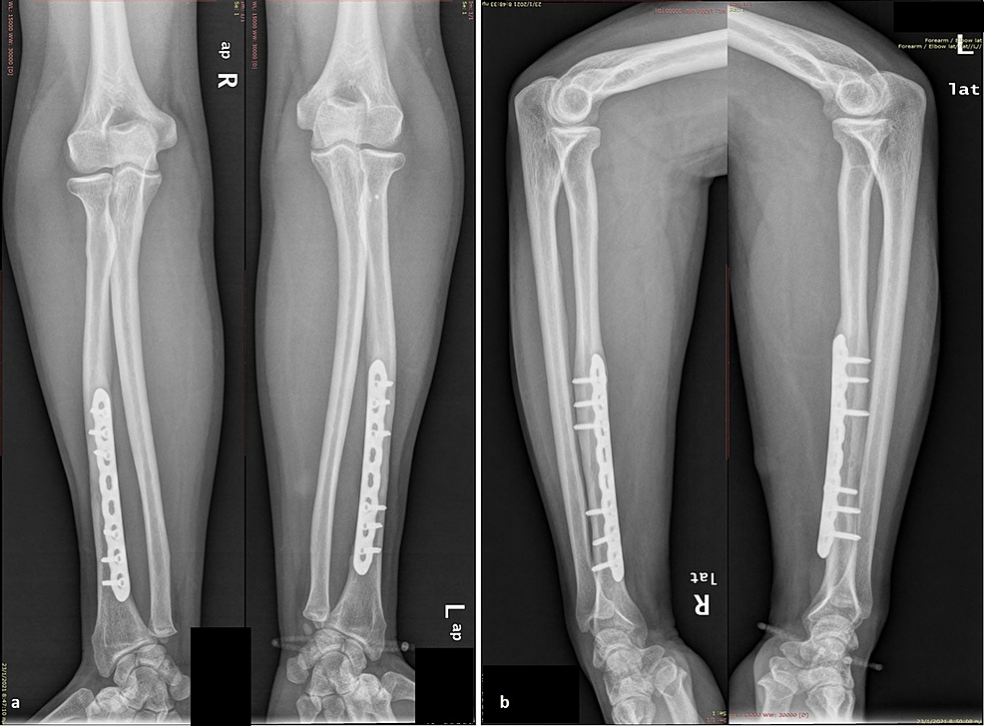
Follow-up radiographs. Anteroposterior (a) and lateral (b) radiographs of both forearms at six years follow-up. There was a union of the radial fractures and stable DRUJs’ joints without evidence of recurrence or ulna migration. The Mayo wrist score was 95 in both limbs. DRUJ: Distal radioulnar joint.

A 31-year-old man sustained injuries in both his forearms as he was riding his bicycle and collided at the side of a car crossing the road ahead of him. At presentation, there was an obvious deformity in both forearms and wrists, marked swelling, and pain on palpation. There was no evidence of neurovascular compromise, compartment syndrome, or other associated injuries. Radiographic evaluation of both forearms revealed bilateral Galeazzi fracture-dislocations with dorsally dislocated ulnar heads (Figure 1). Both radial shaft fractures were simple (AO 22-A2.3), at the same level (Type I: <7.5 cm from the joint line), without severe comminution. Both forearms were splinted temporarily and the patient was transferred to the operating theater nine hours later. Under general anesthesia, antibiotic prophylaxis, and tourniquet usage (up to 250 mm Hg), two surgical teams performed simultaneously open reduction of the radial shaft fracture through an anterior (Henry) approach. Fixation was accomplished in both fractures with an 8-hole, 3.5-mm locking plate (Synthes, Oberdorf, Switzerland). Fluoroscopic examination revealed proper placement of both implants, anatomic reduction of the fractures, correct length of the screws, and reduced DRUJs. The latter were further assessed with several intraoperative stress tests (squeeze test, ulnar pull in the coronal plane using a hook, radial pull test, and DRUJ ballottement test) and were found stable in both forearms. Postoperative radiographic examination revealed proper fracture reduction and stable DRUJs in both forearms (Figure 2). Immobilization in a long forearm cast was applied for a period of four weeks, followed by early passive motion afterward. Active pronation-supination was allowed after six weeks and strengthening exercises after 10 weeks. The patient was followed up regularly on an outpatient basis. At his last follow-up evaluation, six years postoperatively, he was free of pain, had full range of motion, and demonstrated a total Mayo wrist score of 95 (excellent) in both wrists. Radiological examination revealed remodeling of both bones bilaterally, without any evidence of DRUJ subluxation or ulnar migration (Figure 3).

## Discussion

The most important conclusion of the present report is that a successful clinical and radiological outcome can be expected without KW stabilization of the DRUJs in bilateral Galeazzi fracture-dislocations after anatomical open reduction and internal fixation (ORIF) of the radial shaft fractures.

In general, fracture shortening >5 mm, location of the fracture at the distal radial third (7.5 or 10 cm from the joint), widening of the DRUJs on posteroanterior view, displacement of the ulnar relative to the radius on the lateral view, and fracture of the ulnar styloid base, have been proposed as preoperative indicators of DRUJ instability [3,5,6]. Korompilias AV et al. [4] proposed a three-type classification system based on the retrospective evaluation of 95 patients with Galeazzi fractures: Type I was located at the distal third (69 patients), Type II in the middle third (17 patients), and Type III at the proximal third (nine patients). Overall, 40 patients (42%) had DRUJ instability after fixation of the radial fracture and, from those, 37 (92.5%) belonged to type I, as in our case. Tsismenakis T and Tornetta P [11] on the contrary, reported that the radiographic parameters of >5 mm shortening and fractures within 7.5 cm of the joint are not reliable predictors of instability as their positive predictive values were 29% and 19% in respect; the presence of an ulnar styloid fracture may be more helpful (4/7 such cases had an instability). Takemoto R et al. [12] reported that ulnar variance, rather than the absolute distance between the DRUJ and the fracture, is the most reliable predictor of post-fixation persistent DRUJ instability; for each 1 mm of variance away from neutral, there was a 26 % increase in the odds of having DRUJ instability with a 95% CI. In rare instances, the DRUJ joint remains irreducible after fixation of the radial shaft fracture. Yohe NJ et al. in 2019 [6] systematically reviewed the literature and found 17 cases among 12 studies; in dorsally unreduced DRUJs, a block to reduction in most cases (92.3%) was secondary to entrapment of one or more of the extensor tendons, with the remaining cases blocked by fracture fragments. Irreducible volar dislocations due to entrapment of the ulnar head occurred in 17.6% of cases with no tendon entrapment noted.

The key factor for DRUJ transfixation is the intraoperative assessment of its stability, which can be accomplished in various ways. Clinically, the piano key sign, the radius pull test, the ulnocarpal stress test, and the ballottement test, with or without carpal bone stabilization, are the most useful [3,13,14]. Gil JA et al. [15] performed a cadaveric study (11 specimens) assessing three radiographic stress tests-squeeze test, ulnar pull in coronal plane, and simulated DRUJ ballottement test after sequentially sectioning the DRUJ; the squeeze test and simulated DRUJ ballottement test detected a significant increase in diastasis after the foveal attachment of the triangular fibrocartilage complex (TFCC) was sectioned whereas the ulnar pull test in the coronal plane was the most sensitive test for detecting a significant increase in diastasis relative to the intact DRUJ. Finally, El Naga AN et al. [16] proposed the use of the dorsal tangential view (DTV) of the wrist which is used to check the position of the screws after fixation of distal radial fractures. In a cadaveric model, the authors showed that DTV can reliably demonstrate the position of the DRUJ independently of forearm rotation.

Treatment of true Galeazzi fracture-dislocations is operative at least in terms of radial shaft fracture fixation. Alajmi T [1] proposed a treatment algorithm to address DRUJ after radius fixation; when the DRUJ is stable, as in our case, the forearm should be immobilized in supination; if it is unstable, he suggested ulnar styloid fixation, TFCC repair with sutures anchors or/and pinning of DRUJ (with transverse K-wires) with the forearm in supination, followed by postoperative immobilization in supination.

Our comprehensive literature review revealed four cases [7-10] of bilateral Galeazzi fracture-dislocations in the English literature (Table 1). All cases concerned young males who sustained the injury after a high-velocity incident; radial shaft fractures were fixed with plates in all patients and the stability of DRUJ was assessed intraoperatively and found stable in only one case at the right side [7] splinting postoperatively in supination. In that report [10], the left DRUJ was irreducible (due to extensor carpi ulnaris interposition) and managed with open stabilization. In the report of Komura S et al. [7], the left DRJU had acceptable congruity, but the right was found unstable postoperatively (using coronal CT scan images) and later fixed with tension band of the ulnar styloid fracture. In the other two reports [8,9], the DRUJs were unstable after radius ORIF and fixed with a tension band wiring of the ulna styloid fractures [8] and KW fixation and suture repair of both ulnar collateral ligaments and right TFCC in respect. Postoperative immobilization in supination was applied in all patients for a period of 4-6 weeks. The final outcome was good-to-excellent in all reports with the restoration of wrist motion and forearm supination except in one case of persistent DRUJ instability managed with a Darrach procedure upon patient preference [10].

**Table 1:** Literature review table. Details of the current reports of bilateral Galeazzi fracture-dislocations in the English literature. DRUJ: Distal radioulnar joint; LC-DCP: Limited Contact Dynamic Compression Plates; ORIF: Open reduction and internal fixation; ROM: Range of motion; TFCC: Triangular fibrocartilage complex.

Author (year)	Age/Gender	Type of injury	Operative details	Immobilization/Rehabilitation	Follow up (months)	Final outcome
Komura S et al. (2012) [7]	24/Male	Motorcycle accident when collided with a large truck crossing the street ahead of him.	Right radius simple fracture (LC-DCP) and left radius comminuted fracture (ExFix initially, long plate a week later). Persistent DRUJ instability in the right (assessed with postoperative CT) treated a week later with tension band wiring of ulnar styloid fracture.	Splints in both arms for five weeks, early rehabilitation (2w) with active and active-assisted rotation of the forearm from neutral to full supination.	13	Hardware removal at nine months. Right: pronation 75, supination 85 Left: pronation 80, supination 85. Both DRUJs are slightly loose, with mild pain in hyperpronation.
Nanno M et al.(2011) [8]	32/Male	High velocity fall on outstretched hands with forearms in pronation and extension of the wrists and the elbows.	Right radius simple fracture (LC-DCP), unstable DRUJ - ulnar styloid fracture (tension band), scaphoid fracture (Herbert screw), elbow dislocation (closed reduction), left radius comminuted fracture (long LC-DCP plate), unstable DRUJ - ulnar styloid fracture (tension band).	Immobilization for four weeks in neutral forearm rotation and 90^o^ elbow flexion exercise started after four weeks.	84	Hardware removal at 14 months. Right elbow: 0-135^o^ ROM Both wrists and forearms: 60^o ^of palmar and dorsiflexion, 90^o ^pronation and supination.
Chatterjee D (2014) [9]	36/Male	Fall on outstretched hands with the forearm in pronation during horse riding.	Both radial fractures are simply treated with plates. Both DRUJs were unstable (TFCC on the right and both ulnar collateral ligaments were torn and repaired with absorbable sutures followed by K wire fixation of bilateral DRUJ.	Custom-made above elbow braces with a hinge at elbow and wrist immobilized in 30° supination Wrist mobilization in the sagittal plane was begun at one month. Coronal plane movements were initiated after six weeks.	4	Right: supination 80, pronation 80, dorsiflexion 70, palmar flexion 50. Left: supination 85, pronation 80, dorsiflexion 75, palmar flexion 50.
Borens O et al. (2006) [10]	25/Male	He was thrown off his bike after a collision with a stationary motor vehicle.	Right radius fracture type I (Rettig and Raskin's classification) Left radius type II (both ORIF with LC-DCP) Right DRUJ stable after ORIF, left DRUJ irreducible, due to extensor carpi ulnaris interposition (open reduction and 2 KW).	Immobilization of both forearms in supination for six weeks.	20	Persistent DRUJ instability on the left side after KW removal (six weeks); an Adams operation was proposed, but the patient elected a Darrach procedure 12 weeks postoperative At 20 months: Right: pronation 80^ο^, supination 70^ο^ Left: pronation 80^ο^, supination 70^ο^ Wrist motion identical and complete.

## Conclusions

Bilateral Galeazzi fracture-dislocations are rare and complex injuries requiring an attentive assessment of DRUJ stability after anatomical radial shaft plate fixation. Several clinical and fluoroscopic tests should be provided to address DRUJ; if it is stable, a short period of immobilization in supination can lead to a successful clinical and radiological outcome.
